# Antibodies to nodal/paranodal proteins in paediatric immune-mediated neuropathy

**DOI:** 10.1212/NXI.0000000000000763

**Published:** 2020-06-01

**Authors:** Desiree De Simoni, Gerda Ricken, Michael Winklehner, Inga Koneczny, Michael Karenfort, Ulf Hustedt, Ulrich Seidel, Omar Abdel-Mannan, Pinki Munot, Simon Rinaldi, Claudia Steen, Michael Freilinger, Markus Breu, Rainer Seidl, Markus Reindl, Julia Wanschitz, Cinta Lleixà, Günther Bernert, Klaus-Peter Wandinger, Ralf Junker, Luis Querol, Frank Leypoldt, Kevin Rostásy, Romana Höftberger

**Affiliations:** From the Division of Neuropathology and Neurochemistry (D.D.S., G.R., M.W., I.K., R.H.), Department of Neurology, Medical University of Vienna, Austria; Department of Neurology (D.D.S.), University Hospital St. Poelten, Austria; Department of General Pediatrics, Neonatology and Pediatric Cardiology (M.K.), University Children's Hospital, Heinrich Heine University Duesseldorf, Germany; Department of Neuropediatric Rehabilitation (U.H.), Vamed Clinic Hattingen, Germany; Department of Neuropediatrics (U.S.), Charité University, Berlin, Germany; Paediatric Neurology (O.A.-M.), Great Ormond Street Hospital for Children, London, United Kingdom; Dubowitz Neuromuscular Centre (P.M.), Great Ormond Street Hospital for Children, London, United Kingdom; Nuffield Department of Clinical Neurosciences (S.R.), University of Oxford and Oxford University Hospitals NHS Foundation Trust; Department of Paediatric and Adolescent Medicine (C.S.), St Joseph Hospital, Berlin, Germany; Department of Pediatrics and Adolescent Medicine (M.F., M.B., R.S.), Medical University of Vienna, Austria; Department of Neurology (M.R., J.W.), Medical University of Innsbruck, Austria; Neuromuscular Diseases Unit (C.L., L.Q.), Hospital de la Santa Creu i Sant Pau, Universitat Autónoma de Barcelona, Spain; SMZ Süd (G.B.), Kaiser-Franz Josef Hospital with Gottfried von Preyer Children Hospital, Vienna, Austria; Institute of Clinical Chemistry (K.-P.W., R.J., F.L.), University Hospital Schleswig-Holstein, Kiel/Lübeck, Germany; Department of Neurology (F.L.), University Hospital Schleswig-Holstein, Kiel, Germany; and Department of Pediatric Neurology (K.R.), Witten/Herdecke University, Children's Hospital Datteln, Germany.

Patients with nodal/paranodal antibodies represent a specific subgroup of inflammatory peripheral neuropathies, whose clinical presentation with a prolonged subacute phase, additional symptoms such as ataxia and tremor, and poor treatment response to IV immunoglobulin (IVIG) often differs from classic Guillain-Barré syndrome (GBS) or chronic inflammatory demyelinating polyneuropathy (CIDP).^1^

Previous studies on nodo/paranodopathies mainly focused on adult patients, whereas the clinical spectrum of pediatric patients is less well established. We reviewed the clinical presentation of 54 children with GBS (n = 42) and CIDP (n = 12) and retrospectively screened for antibodies against neurofascin155 (NF155), NF186, NF140, contactin-1 (CNTN1), contactin-associated protein1 (CASPR1), and glycine-receptor (GlyR) using cell-based assays^2,3^; 1 patient was additionally tested with CNTN1-ELISA.^4^ All cases with sufficient serum were tested for ganglioside-IgG-, IgA-, and IgM-antibodies against GM1 (n = 42), GD1a (n = 18), GD1b (n = 23), and GQ1b (n = 21).^5^ Clinical and paraclinical information of all patients is summarized in the table. The study was approved by the ethics committee (EK1773/2016).

**Table:**
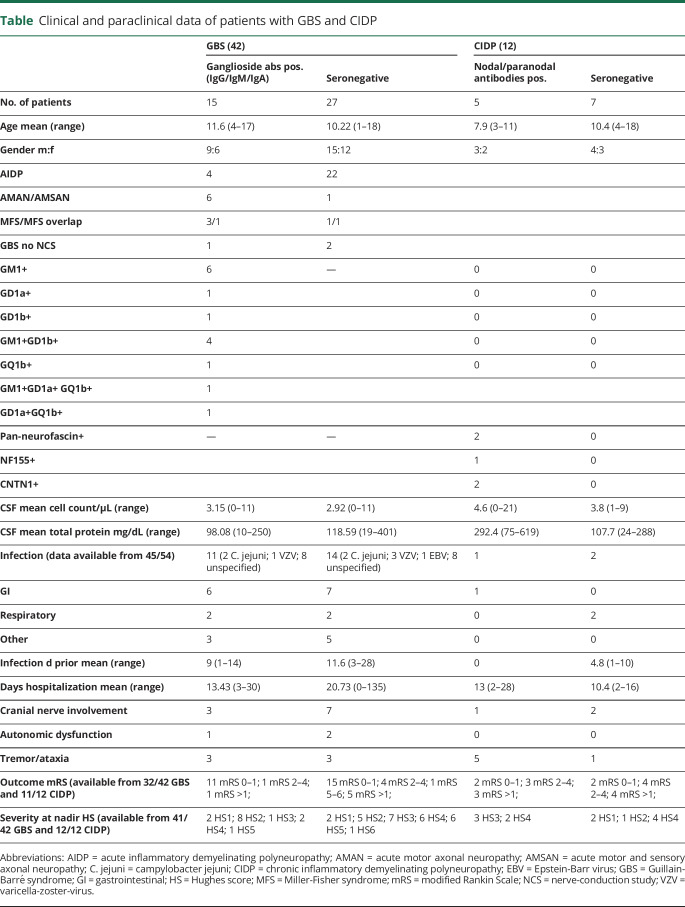
Clinical and paraclinical data of patients with GBS and CIDP

## Children with classic GBS

Of 42 children with GBS, 26 were classified as acute inflammatory demyelinating polyneuropathy (AIDP), 7 as acute motor/motor-sensory axonal neuropathy (AMAN/AMSAN) by nerve conduction velocity according to Hadden criteria,^6^ 4 as Miller-Fisher syndrome (MFS), and 2 as MFS/GBS overlap. Three patients with GBS could not be classified because of lack of nerve-conduction studies. In 25 of 35 patients (71.4%), an infection was reported within 4 weeks before symptom onset (13 gastrointestinal, 4 respiratory, and 8 unspecified). Eight patients had IgG-ganglioside antibodies (19.0%), 6 IgM (14.2%), and 1 IgA (2.4%). Nodal/paranodal antibodies were not detected. Patients with AMAN/AMSAN (5/7 with reported infection: 1 campylobacter jejuni, 1 varicella-zoster virus, and 3 unspecified) were more often ganglioside antibody positive (6/7) than patients with AIDP (4/26; likelihood ratio 12.419) or MFS (2/4).

## Children with nodal/paranodal antibodies

Five of 12 children, who met the EFNS/PNS criteria for CIDP, had nodal/paranodal antibodies: 2 pan-neurofascin (NF155/NF186/140 triple positive), 1 NF155, and 2 CNTN1-antibodies. The IgG-subclass distribution was determined by flow cytometry analysis.^7^ IgG4 was the predominant subclass in all patients and ranged from 75% to 100%. In addition, 1 patient with pan-neurofascin-antibodies tested positive for GlyR-antibodies but did not develop stiff-person syndrome or progressive encephalomyelitis with rigidity, and the significance of this finding needs further investigation. The mean age was 7.9 years (range 3–11), and the male:female ratio was 3:2. The median duration of hospitalization was 13 days (range 2–28). One pan-neurofascin-patient was initially diagnosed as GBS and reclassified as CIDP during disease course, the other patients had a chronic onset with slow progression over months or years. One child had a gastrointestinal infection before symptom onset. One CNTN1-patient showed cranial nerve involvement and optic neuritis during disease course. All children had ataxia, 4 neuropathic pain (all except 1 pan-neurofascin), and 3 (2 CNTN1, and 1 pan-neurofascin) tremor. At the peak of disease, 3 children needed a walking aid (Hughes 3) and 2 were bedridden (Hughes 4). None of the children had renal dysfunction. The mean CSF white cell count was 4.6 μL (range 0–21), and the mean CSF protein was 292.4 mg/dL (range 75–619).

The mean time of follow-up was 32 months (range 17–57). The 2 CIDP patients with pan-neurofascin-antibodies initially showed no or only partial response to IVIG and therefore received corticosteroids, 1 along with plasma exchange and the other with mycophenolate. Both recovered only very slowly over up to 4 years with a modified Rankin Scale (mRS) score of 1 at the last follow-up. The NF155-patient did not respond to IVIG and corticosteroids and subsequently received immunoadsorption and rituximab, leading to significant clinical improvement. After 8 months, he relapsed in association with normalization of the CD19/20 ratio and again rapidly improved after another dose of rituximab, with a mRS score of 2 at the last follow-up. One patient with CNTN1-antibodies worsened despite monthly IVIG and corticosteroids given over 4 months. After treatment was switched to rituximab, he improved rapidly in the following weeks and remained stable since then. The second child with CNTN1-antibodies showed only partial response to IVIG with relapses in conjunction with infections. This child improved significantly after rituximab application with a mRS score of 2 at the last follow-up.

In summary, our study demonstrates that nodal/paranodal antibodies occur in a subgroup of paediatric patients with CIDP, but not GBS. Children with AMAN/AMSAN frequently have ganglioside antibodies. Children with CIDP and atypical/prolonged disease course with high Hughes score (>2), sensory ataxia, prominent neuropathic pain, and tremor may have nodal/paranodal antibodies. These patients often do not sufficiently respond to IVIG, whereas in our case series, rituximab led to prompt improvement in 3 children. Optimal treatment strategies for children with nodal/paranodal antibodies have to be further determined in larger studies.
